# Midodrine hydro­chloride Form A, C_12_H_19_N_2_O_4_^+^·Cl^−^

**DOI:** 10.1107/S2056989026004810

**Published:** 2026-05-15

**Authors:** Jacob K. Salazar, James A. Kaduk, Anja Dosen, Thomas N. Blanton

**Affiliations:** a North Central College, Department of Chemistry 131 S Loomis St Naperville IL 60540 USA; b North Central College, Department of Physics 131 S Loomis St Naperville IL 60540 USA; c Illinois Institute of Technology, Department of Chemistry, 3101 S. Dearborn St., Chicago, IL 60616, USA; d ICDD, 12 Campus Blvd., Newtown Square, PA 19073-3273, USA; Harvard University, USA

**Keywords:** powder diffraction, midodrine, ProAmatine®, Rietveld refinement, density functional theory

## Abstract

The crystal structure of midodrine hydro­chloride Form A (systematic name: {[2-(2,5-di­meth­oxy­phen­yl)-2-hy­droxy­eth­yl]carbamo­yl}methanaminium chloride, C_12_H_19_N_2_O_4_^+^·Cl^−^) has been solved and refined using synchrotron X-ray powder diffraction data, and optimized using density functional theory techniques. Midodrine hydro­chloride Form A crystallizes in space group *P2_1_/c* (#14). The crystal structure is characterized by layers perpendicular to the *c*-axis direction. The Cl anions reside in the center of the layer. Hydrogen bonds are prominent in the structure. Each of the three H on the protonated N acts as a donor – one to the carbonyl group and the other two to the Cl ion. The hydroxyl group and the amide also act as donors to Cl. Considering the classical hydrogen bonds, the Cl ion is four-coordinate.

## Abstract 2

The crystal structure of midodrine hydro­chloride Form A has been solved and refined using synchrotron X-ray powder diffraction data, and optimized using density functional theory techniques.

## 1. Chemical context

Midodrine hydro­chloride (marketed as ProAmatine, among others) is used to treat hypotension (low blood pressure) and urinary incontinence. In particular, midodrine HCl treats symptomatic low blood pressure upon standing from a sitting or laying down position. The systematic name (CAS Registry Number 43218-56-0) is 2-amino-*N*-[2-(2,5-di­meth­oxy­phen­yl)-2-hy­droxy­eth­yl]acetamide hydro­chloride.
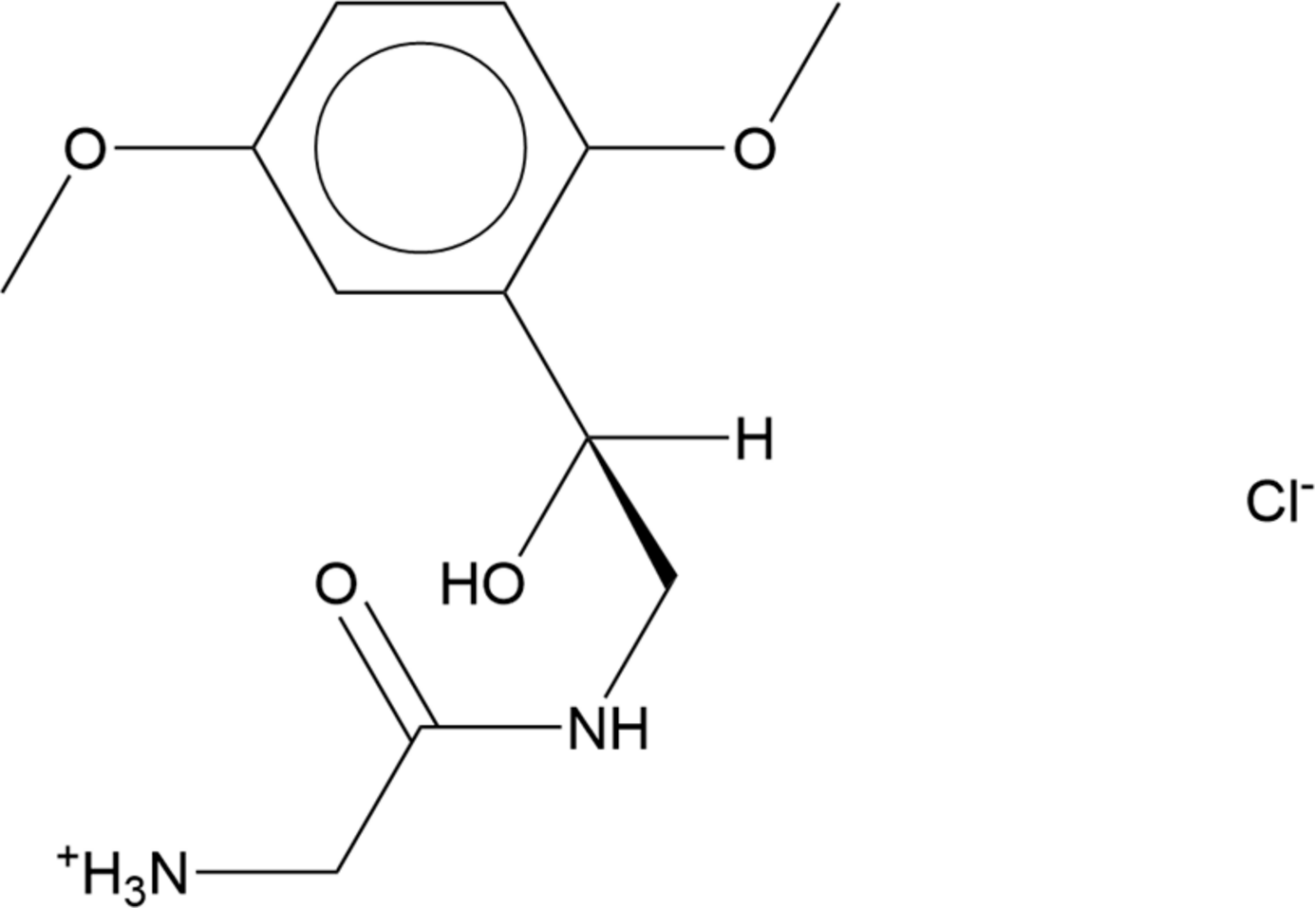


A process for preparing midodrine hydro­chloride has been claimed in US Patent Application US 2022/0144754 A1 (Singh *et al.*, 2022 ▸; Cadila Healthcare Ltd.), and powder diffraction data labeled as Form A are provided but no crystal structure was reported.

This work was carried out as part of a project (Kaduk *et al.*, 2014 ▸) to determine the crystal structures of large-volume commercial pharmaceuticals, and includes high-quality powder diffraction data for them in the Powder Diffraction File (Kabekkodu *et al.*, 2024 ▸).

## 2. Structural commentary

**Figure 1:**
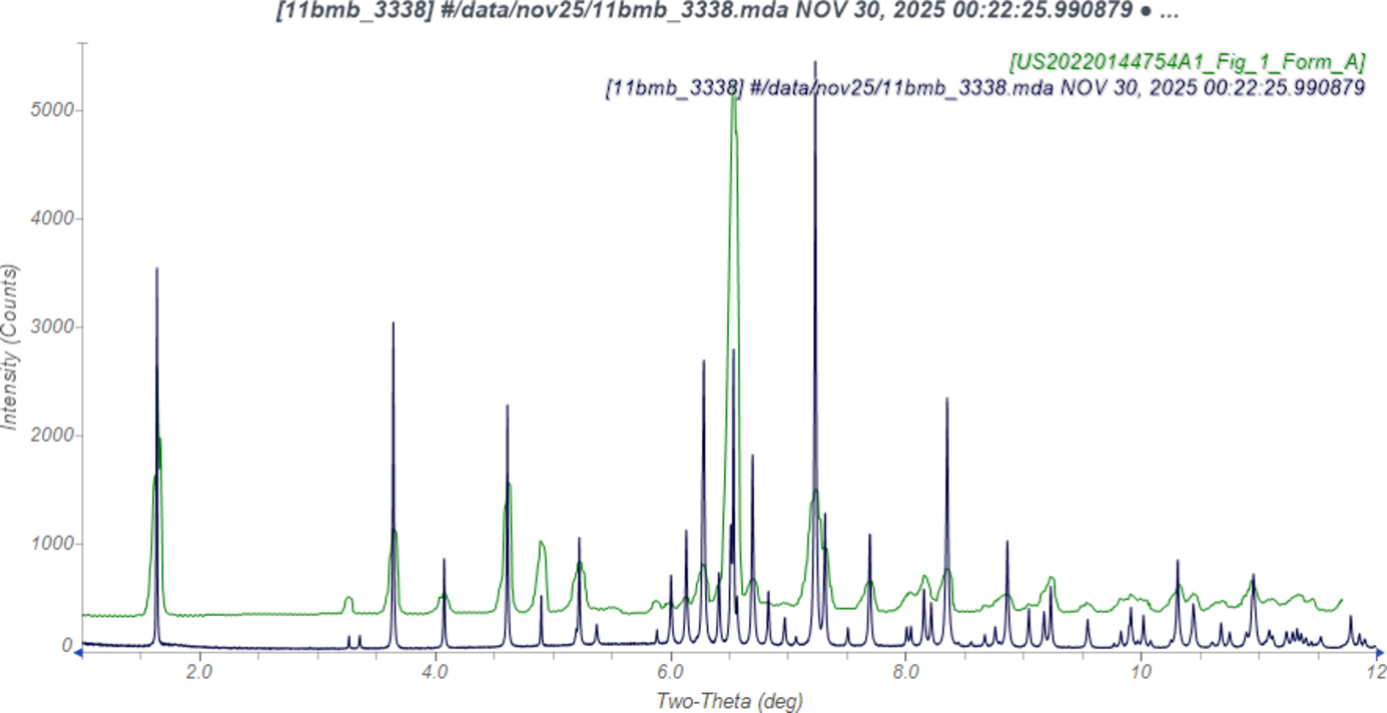
Comparison of the synchrotron pattern from this study of midodrine hydro­chloride (black) to that reported for Form A by Singh *et al.* (2022 ▸; green). The patent pattern (measured using Cu *K*_α_ radiation) was digitized using *UN-SCAN-IT* (Silk Scientific, 2013 ▸) and converted to the synchrotron wavelength of 0.4687342 Å using *JADE Pro* (MDI, 2026 ▸). Image generated using *JADE Pro* (MDI, 2026 ▸).

The synchrotron pattern of midodrine hydro­chloride is similar enough to that reported by Singh *et al.* (2022 ▸) for Form A (Fig. 1 ▸) to conclude that they represent the same material. The patent pattern exhibits small displacement/transparency peak position error, as well as significant preferred orientation.

**Figure 2:**
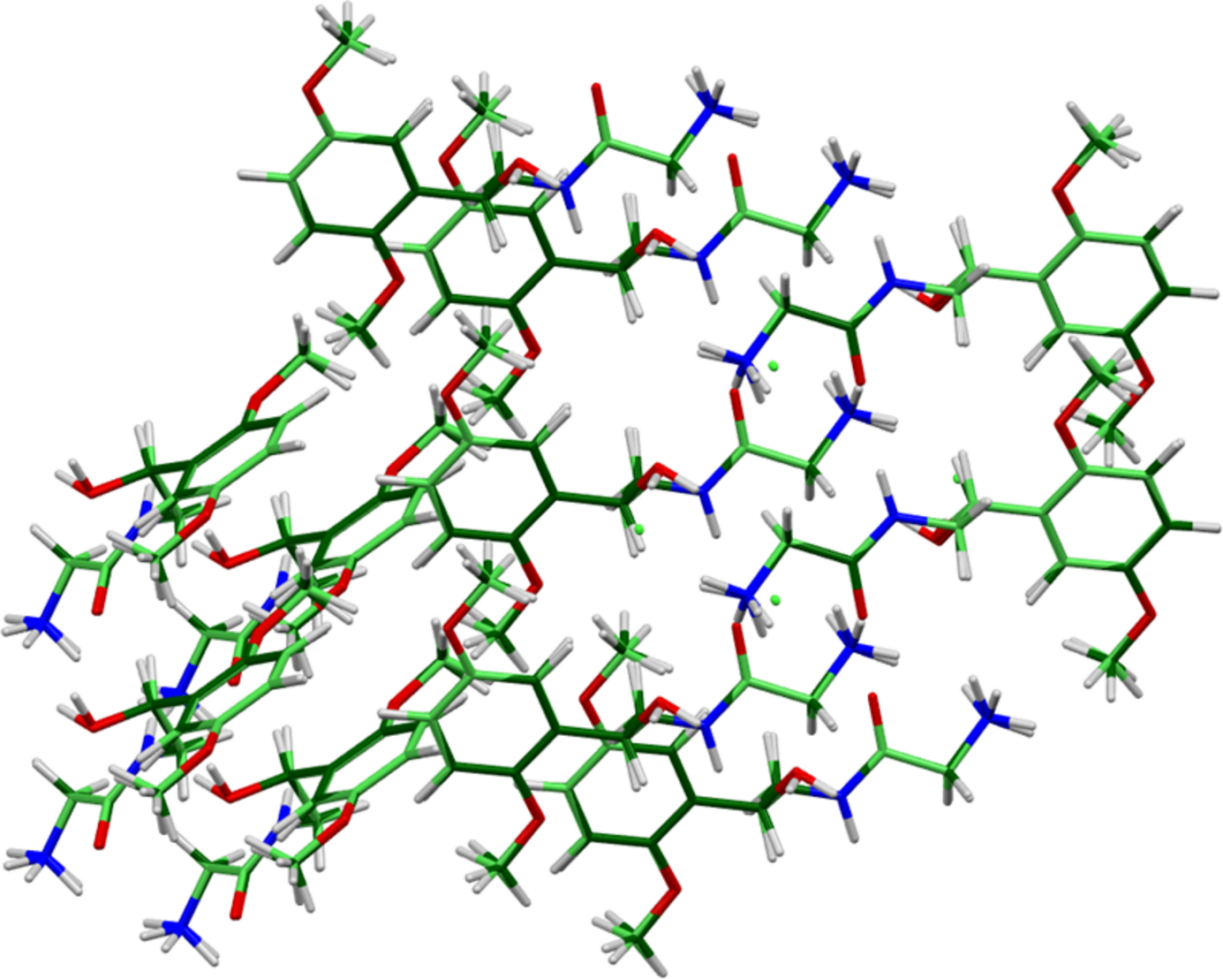
Comparison of the Rietveld-refined (colored by atom type) and *VASP*-optimized (pale green) structures of midodrine hydro­chloride Form A, calculated using the *Mercury* CSD-Materials/Search/Crystal Packing Similarity tool. The root-mean-square Cartesian displacement is 0.050 Å. Image generated using *Mercury* (Macrae *et al.*, 2020 ▸).

**Figure 3:**
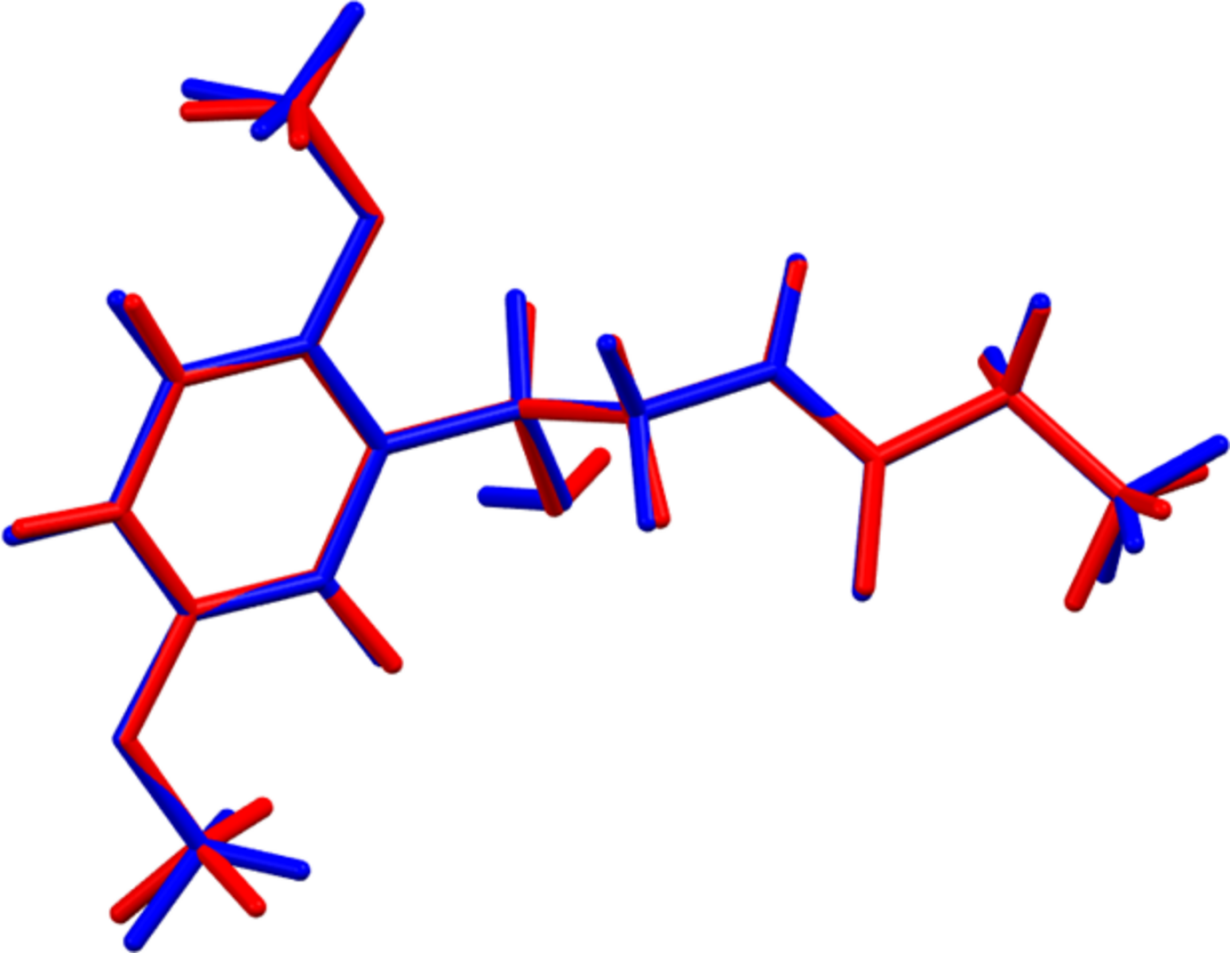
Comparison of the refined structure of the cation in midodrine hydro­chloride Form A (red) to the *VASP*-optimized structure (blue). The comparison was generated using the *Mercury* Calculate/Mol­ecule Overlay tool; the r.m.s. deviation is 0.042 Å. Image generated using *Mercury* (Macrae *et al.*, 2020 ▸).

**Figure 4:**
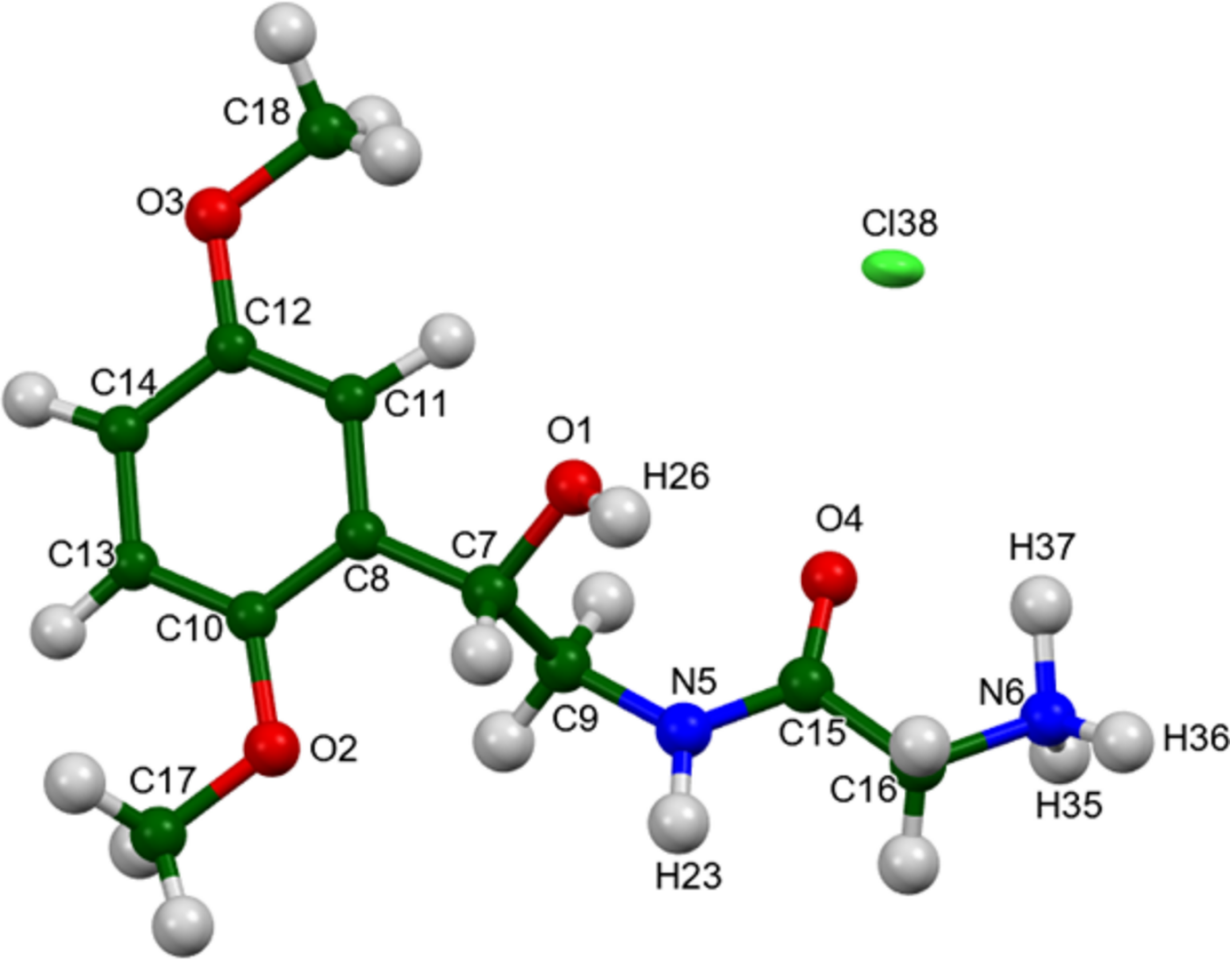
The asymmetric unit of midodrine hydro­chloride Form A, with the atom numbering. The atoms are represented by 50% probability spheroids/ellipsoids. Image generated using *Mercury* (Macrae *et al.*, 2020 ▸).

The root-mean-square deviation of the non-H atoms in the Rietveld-refined and *VASP*-optimized structures of midodrine hydro­chloride Form A, calculated using the *Mercury* CSD-Materials/Search/Crystal Packing Similarity tool (Macrae *et al.*, 2020 ▸) is 0.050 Å (Fig. 2 ▸); the structures are essentially identical. The root-mean-square Cartesian displacement of the non-H atoms in the refined and optimized structures of the cation, calculated using the *Mercury* Calculate/Mol­ecule Overlay tool, is 0.042 Å (Fig. 3 ▸). The absolute position difference of the Cl is 0.033 Å. The agreements are within the normal range for correct structures (van de Streek & Neumann, 2014 ▸). The asymmetric unit is illustrated in Fig. 4 ▸. The remaining discussion will emphasize the *VASP*-optimized structure.

All of the bond distances, bond angles, and torsion angles (Table 1 ▸) fall within the normal ranges indicated by a *Mercury* Mogul Geometry check (Macrae *et al.*, 2020 ▸). Quantum chemical geometry optimization of the isolated midodrine cation (DFT/B3LYP/6-31G*/water) using *Spartan ’24* (Wavefunction, 2025 ▸) indicated that the observed conformation is 4.4 kcal mol^−1^ higher in energy than a local minimum, which has an essentially identical conformation (r.m.s. deviation = 0.066 Å). The global minimum-energy conformation (MMFF force field) is 98.1 kcal mol^−1^ lower in energy, but is unreasonably folded on itself to form intra­molecular hydrogen bonds. Inter­molecular inter­actions are important in determining the solid-state conformation.

## 3. Supra­molecular features

**Figure 5:**
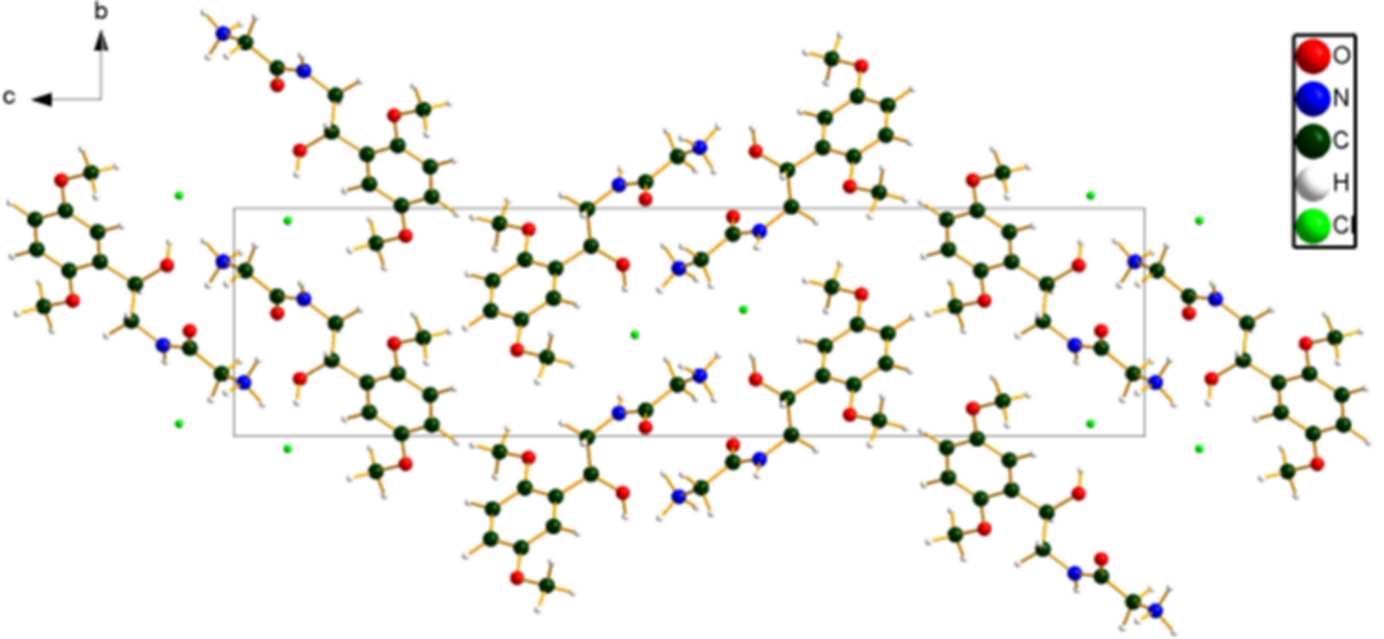
Crystal structure of midodrine hydro­chloride Form A, viewed down the *a*-axis. Image generated using *DIAMOND* (Crystal Impact, 2025 ▸).

The crystal structure (Fig. 5 ▸) is characterized by layers perpendicular to the *c*-axis direction. The Cl anions reside in the center of the layer. The *Mercury* Aromatics Analyser indicates three moderate inter­actions (*d* = 5.18, 5.18, and 6.08 Å), which include both slipped stacking and end-face inter­actions. The mean plane of the aromatic rings is approximately (3,4,10).

Analysis of the contributions to the total crystal energy of the structure using the Forcite module of *Materials Studio* (Dassault Systèmes, 2025 ▸) indicated that the intra­molecular energy is dominated by angle distortion terms. The inter­molecular energy is small and dominated by van der Waals repulsions, which in this force field based analysis include hydrogen bonds. The hydrogen bonds are better discussed using the results of the DFT calculation.

**Figure 6:**
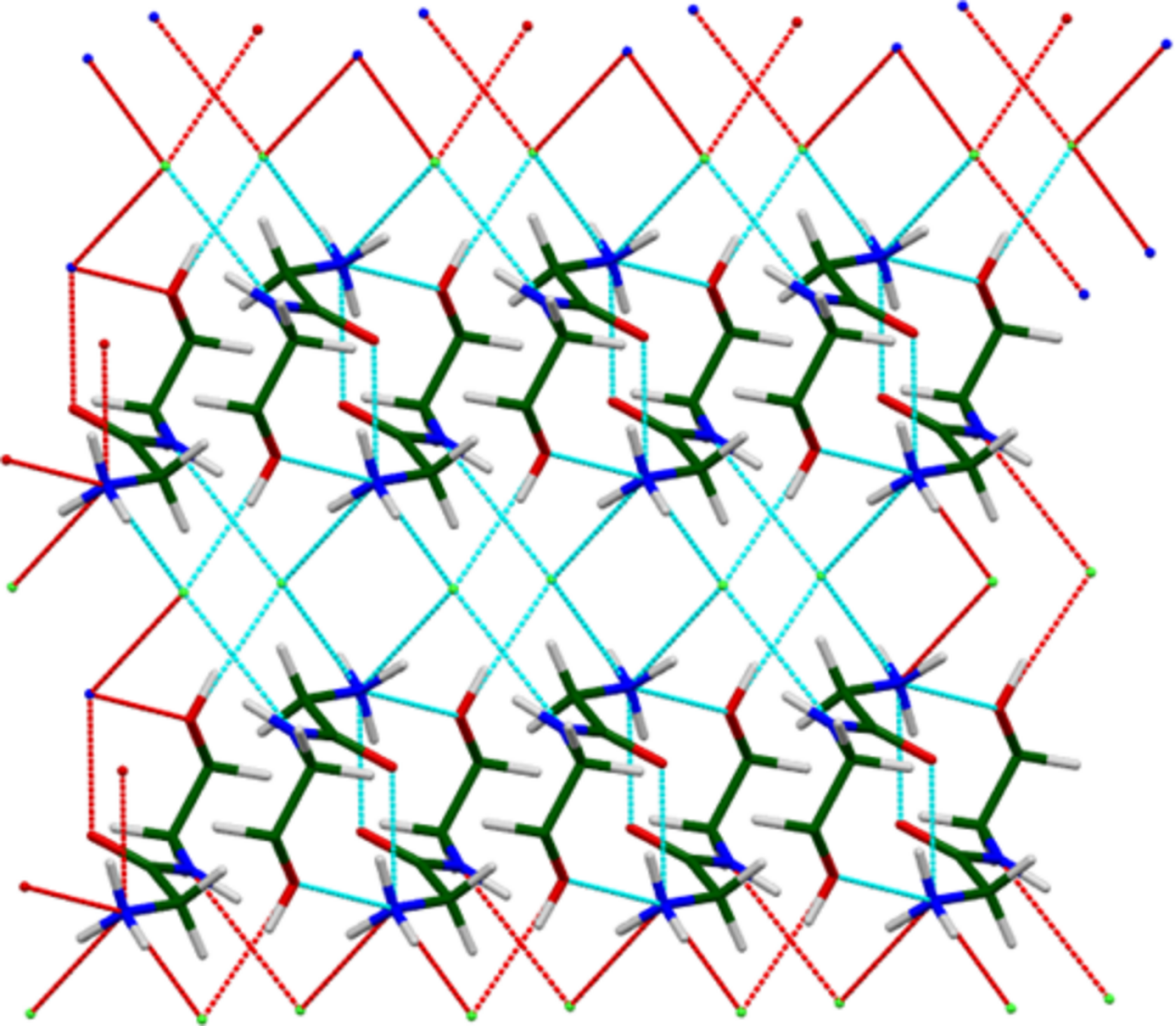
The hydrogen bonding pattern in the layers of midodrine hydro­chloride Form A. Image generated using *Mercury* (Macrae *et al.*, 2020 ▸).

Hydrogen bonds (Table 2 ▸) are prominent in the structure. Each of the three H on the protonated N6 acts as a donor – one to the carbonyl group O4 and the other two to Cl38. The energy of the N—H⋯O hydrogen bond was calculated using the correlation of Wheatley & Kaduk (2019 ▸). The hydroxyl group O1—H26 also acts as a donor to Cl38. The energy of the O—H⋯Cl bond was calculated using the correlation of Kaduk (2002 ▸). The amide N5—H23 also acts as a donor to Cl38. Considering the classical hydrogen bonds, the Cl is four-coordinate. These hydrogen bonds result in rings and chains, with graph sets (Etter, 1990 ▸; Bernstein *et al.*, 1995 ▸; Motherwell *et al.*, 2000 ▸) 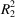
(10), 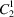
(7), 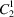
(10), 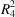
(20), and larger features. The result is a complex network of hydrogen bonds in the center of the layers (Fig. 6 ▸). C—H⋯Cl and C—H⋯O hydrogen bonds also contribute to the lattice energy.

**Figure 7:**
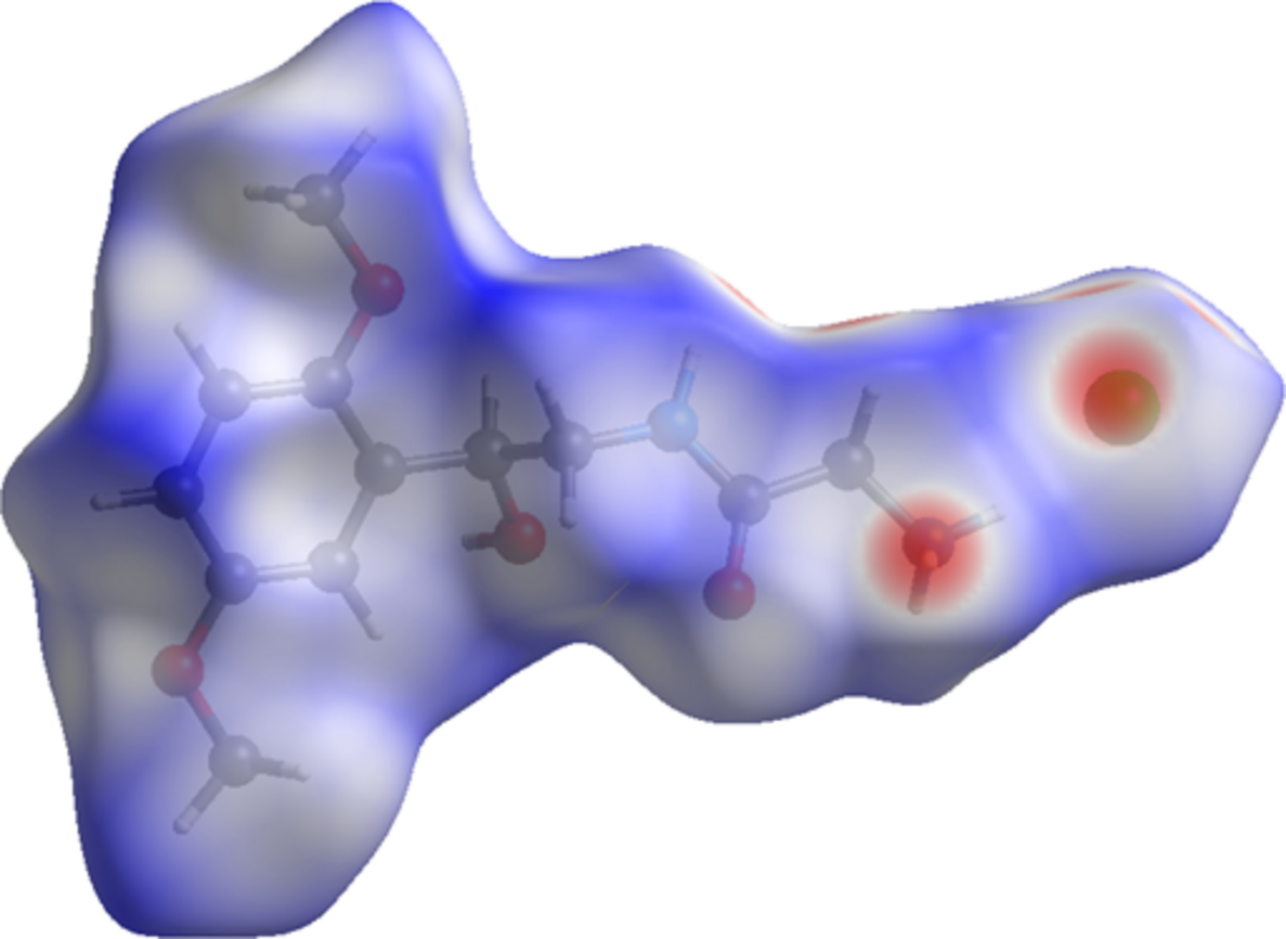
The Hirshfeld surface of midodrine hydro­chloride Form A. Inter­molecular contacts longer than the sums of the van der Waals radii are colored blue, and contacts shorter than the sums of the radii are colored red. Contacts equal to the sums of radii are white. Image generated using *CrystalExplorer* (Spackman *et al.*, 2021 ▸).

The volume enclosed by the Hirshfeld surface of midodrine hydro­chloride Form A (Fig. 7 ▸, Hirshfeld, 1977 ▸, Spackman *et al.*, 2021 ▸) is 343.78 Å^3^, 97.74% of 1/4 of the unit-cell volume. The packing density is thus typical. The close contacts (red in Fig. 7 ▸) involve the hydrogen bonds. The volume/non-hydrogen atom is normal, at 18.5 Å^3^.

The Bravais–Friedel–Donnay–Harker (Bravais, 1866 ▸; Friedel, 1907 ▸; Donnay & Harker, 1937 ▸) algorithm suggests that we might expect platy morphology for midodrine hydro­chloride Form A, with {002} as the major faces. A 2nd-order spherical harmonic model for preferred orientation was included. The texture index was 1.003, indicating that the preferred orientation was negligible in this rotated capillary specimen.

## 4. Database survey

A reduced cell search in the Cambridge Structural Database (CSD 2026.1.0; Groom *et al.*, 2016 ▸) yielded 16 hits, but no structures of midodrine or its derivatives.

## 5. Synthesis and crystallization

Midodrine hydro­chloride was a commercial reagent, purchased from TargetMol (Batch #150940), and was used as-received.

## 6. Refinement

Crystal data, data collection and structure refinement details are summarized in Table 3 ▸. The white powder was packed into a 1.5 mm diameter Kapton capillary, and rotated during the measurement at ∼50 Hz. The powder pattern was measured at 295 K at beam line 11-BM (Lee *et al.*, 2008 ▸; Wang *et al.*, 2008 ▸; Antao *et al.*, 2008 ▸) of the Advanced Photon Source at Argonne National Laboratory using a wavelength of 0.4687342 Å from 0.5–50° 2θ with a step size of 0.001° and a counting time of 0.1 sec/step. The high-resolution powder diffraction data were collected using twelve silicon crystal analyzers that allow for high angular resolution, high precision, and accurate peak positions. A mixture of silicon (NIST SRM 640c) and alumina (NIST SRM 676a) standards (ratio Al_2_O_3_:Si = 2:1 by weight) was used to calibrate the instrument and refine the monochromatic wavelength used in the experiment.

The pattern was indexed on a primitive monoclinic unit cell with *a* = 5.17847, *b* = 8.25254, *c* = 32.94869 Å, *β* = 92.757°, *V* = 1406.4 Å^3^, and *Z* = 4 using *N-TREOR* as incorporated into *EXPO2014* (Altomare *et al.*, 2013 ▸). The suggested space group was *P*2_1_/*c*, which was confirmed by the successful solution and refinement of the structure.

The mol­ecular structure of midodrine was downloaded from PubChem (Kim *et al.*, 2023 ▸) as Conformer3D_COMPOUND_CID_4195.sdf. It was converted to a *.mol2 file using *Mercury* (Macrae *et al.*, 2020 ▸), and to a Fenske–Hall *Z*-matrix using *OpenBabel* (O’Boyle *et al.*, 2011 ▸). The structure was solved by parallel tempering techniques as implemented in *FOX* (Favre-Nicolin & Černý, 2002 ▸) using a midodrine mol­ecule and a Cl atom as fragments, and by Monte Carlo simulated annealing techniques as implemented in *EXPO2014* (Altomare *et al.*, 2013 ▸) and *DASH* (David *et al.*, 2006 ▸). All three programs yielded equivalent structures. The *FOX* structure was selected for refinement. H37 was added to N6 using Mercury.

Rietveld refinement was carried out using *GSAS-II* (Toby & Von Dreele, 2013 ▸). Only the 1.5–28.0° portion of the pattern was included in the refinements (*d_min_* = 0.969 Å). All non-H bond distances and angles were subjected to restraints, based on a *Mercury*/Mogul Geometry Check (Sykes *et al.*, 2011 ▸; Bruno *et al.*, 2004 ▸). The Mogul average and standard deviation for each qu­antity were used as the restraint parameters. The phenyl ring was restrained to be planar. The restraints contributed 1.5% to the overall *χ^2^*. The hydrogen atoms were included in calculated positions, which were recalculated during the refinement using *Materials Studio* (Dassault Systèmes, 2025 ▸). The *U*_iso_ of the non-H atoms were grouped by chemical similarity. The *U_iso_* of the H atoms were fixed at 1.2 × the *U*_iso_ of the heavy atom to which they are attached. The Cl was refined anisotropically. The peak profiles were described using the generalized microstrain model (Stephens, 1999 ▸). The background was modeled using a six-term shifted Chebyshev polynomial, with peaks at 1.58 and 5.56° to model the scattering from the Kapton capillary and any amorphous component of the sample.

**Figure 8:**
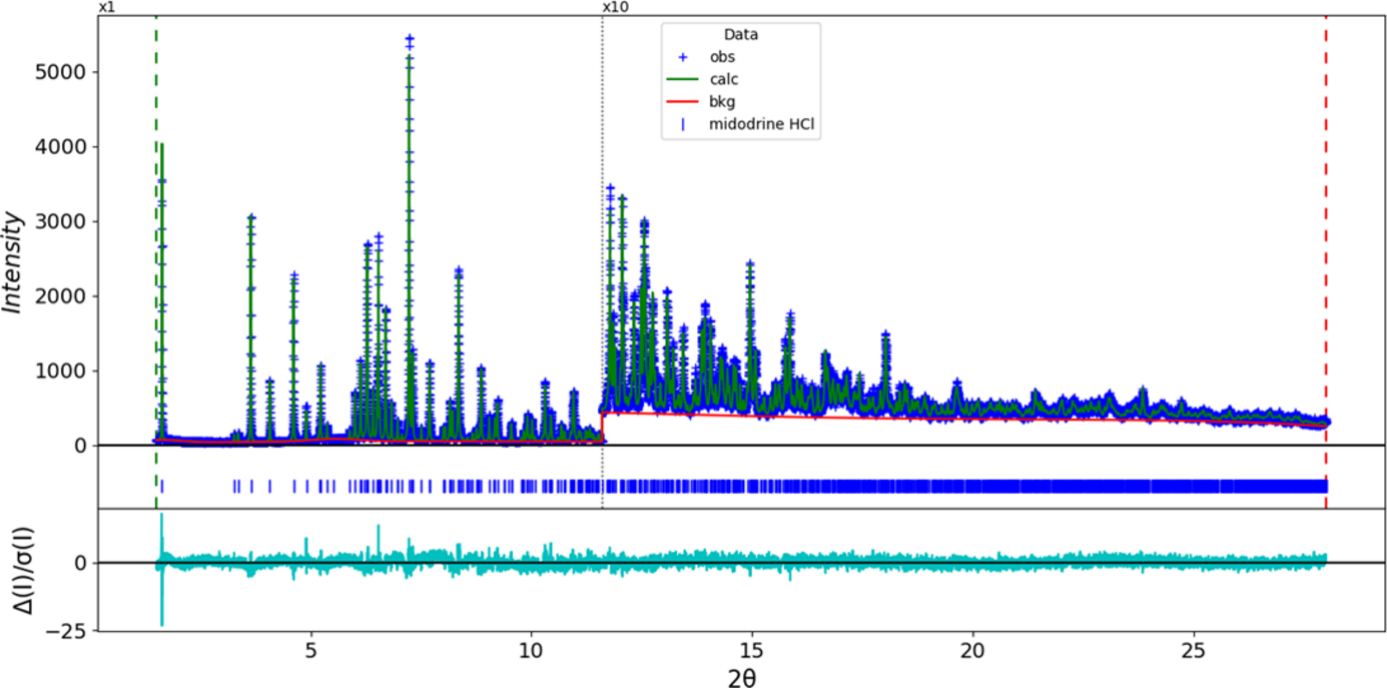
The Rietveld plot for midodrine hydro­chloride Form A. The blue crosses represent the observed data points, and the green line is the calculated pattern. The cyan curve is the normalized error plot, and the red line is the background curve. The blue tick marks indicate the peak positions. The vertical scale has been multiplied by a factor of 10× for 2θ > 11.6°.

The final refinement of 96 variables using 26,501 observations and 42 restraints yielded the residuals *R*_wp_ = 0.06154 and GOF = 1.42. The largest peak (0.77 Å from O3) and hole (0.37 Å from O3) in the difference-Fourier map were 0.17 (4) and −0.17 (4) e Å^−3^, respectively. The final Rietveld plot is shown in Fig. 8 ▸. The largest features in the normalized error plot are in the shapes of some of the strong low-angle peaks.

The crystal structure of midodrine hydro­chloride Form A was optimized (fixed experimental unit cell) with density functional theory techniques using *VASP* (Kresse & Furthmüller, 1996 ▸) through the *MedeA* graphical inter­face (Materials Design, 2024 ▸). The calculation was carried out on 32 cores of a 144-core (768 Gb memory) HPE Superdome Flex 280 Linux server at North Central College. The calculation used the GGA-PBE functional, a plane wave cutoff energy of 400.0 eV, and a *k*-point spacing of 0.5 Å^−1^ leading to a 3 × 2 × 1 mesh, and took ∼2.6 h. Single-point density functional theory calculations (fixed experimental cell) and population analysis were carried out using *CRYSTAL23* (Erba *et al.*, 2023 ▸) and *CRYSTAL17* (Dovesi *et al.*, 2018 ▸). The basis sets for the H, C, N and O atoms in the calculation were those of Gatti *et al.* (1994 ▸), and that for Cl was from Peintinger *et al.* (2013 ▸). The calculations were run on a 3.5 GHz PC using 8 *k*-points and the B3LYP functional, and took ∼1.8 hr. The powder pattern has been submitted to ICDD for inclusion in the Powder Diffraction File™ (PDF®).

## Supplementary Material

Crystal structure: contains datablock(s) midodrine, midodrine_midodrine_VASP. DOI: 10.1107/S2056989026004810/oi2037sup1.cif

Supporting information file. DOI: 10.1107/S2056989026004810/oi2037midodrinesup2.cml

CCDC references: 2552708, 2552707

Additional supporting information:  crystallographic information; 3D view; checkCIF report

## Figures and Tables

**Table 1:** Selected geometric parameters (Å, °) for midodrine[Chem scheme1]

O1—C7	1.437 (2)	C15—O4	1.224 (2)
O1—H26	0.989 (2)	C15—N5	1.329 (2)
O2—C10	1.3741 (17)	C15—C16	1.5115 (19)
O2—C17	1.427 (3)	C16—N6	1.467 (3)
O3—C12	1.3814 (19)	C16—C15	1.5115 (19)
O3—C18	1.425 (3)	C16—H27	1.085 (3)
O4—C15	1.224 (2)	C16—H28	1.125 (3)
N5—C9	1.451 (2)	C17—O2	1.427 (3)
N5—C15	1.329 (2)	C17—H29	1.060 (3)
N5—H23	1.047 (2)	C17—H30	1.115 (3)
N6—C16	1.467 (3)	C17—H31	1.100 (3)
N6—H35	1.072 (3)	C18—O3	1.425 (3)
N6—H36	1.070 (2)	C18—H32	1.142 (3)
N6—H37	1.015 (3)	C18—H33	1.067 (3)
C7—O1	1.437 (2)	C18—H34	1.089 (3)
C7—C8	1.5181 (10)	H19—C7	1.118 (3)
C7—C9	1.519 (3)	H20—C9	1.116 (3)
C7—H19	1.118 (3)	H21—C9	1.121 (3)
C8—C7	1.5181 (10)	H22—C11	1.0811 (19)
C8—C10	1.3914 (18)	H23—N5	1.047 (2)
C8—C11	1.4010 (18)	H24—C13	1.119 (2)
C9—N5	1.451 (2)	H25—C14	1.1036 (19)
C9—C7	1.519 (3)	H26—O1	0.989 (2)
C9—H20	1.116 (3)	H26—Cl38^i^	2.1733 (10)
C9—H21	1.121 (3)	H27—C16	1.085 (3)
C10—O2	1.3741 (17)	H28—C16	1.125 (3)
C10—C8	1.3914 (18)	H29—C17	1.060 (3)
C10—C13	1.391 (2)	H30—C17	1.115 (3)
C11—C8	1.4010 (18)	H31—C17	1.100 (3)
C11—C12	1.3917 (18)	H32—C18	1.142 (3)
C11—H22	1.0811 (19)	H33—C18	1.067 (3)
C12—O3	1.3814 (19)	H34—C18	1.089 (3)
C12—C11	1.3917 (18)	H35—N6	1.072 (3)
C12—C14	1.383 (2)	H35—Cl38^ii^	2.1656 (10)
C13—C10	1.391 (2)	H36—N6	1.070 (2)
C13—C14	1.378 (2)	H36—Cl38^iii^	2.0529 (10)
C13—H24	1.119 (2)	H37—N6	1.015 (3)
C14—C12	1.383 (2)	Cl38—H26^i^	2.1733 (10)
C14—C13	1.378 (2)	Cl38—H35^ii^	2.1656 (10)
C14—H25	1.1036 (19)	Cl38—H36^iv^	2.0529 (10)
			
C7—O1—H26	104.5 (2)	C12—C11—H22	122.58 (19)
C10—O2—C17	118.16 (15)	O3—C12—C11	123.4 (2)
C12—O3—C18	118.2 (2)	O3—C12—C14	116.5 (2)
C9—N5—C15	123.69 (18)	C11—C12—C14	120.08 (12)
C9—N5—H23	117.7 (2)	C10—C13—C14	120.27 (14)
C15—N5—H23	117.5 (2)	C10—C13—H24	120.83 (18)
C16—N6—H35	108.7 (2)	C14—C13—H24	118.9 (2)
C16—N6—H36	109.0 (2)	C12—C14—C13	120.11 (13)
H35—N6—H36	104.9 (2)	C12—C14—H25	119.3 (2)
C16—N6—H37	111.8 (3)	C13—C14—H25	120.6 (2)
H35—N6—H37	111.9 (2)	O4—C15—N5	124.03 (18)
H36—N6—H37	110.3 (2)	O4—C15—C16	120.44 (17)
O1—C7—C8	111.60 (16)	N5—C15—C16	115.28 (18)
O1—C7—C9	107.8 (2)	N6—C16—C15	110.65 (17)
C8—C7—C9	111.2 (2)	N6—C16—H27	107.5 (3)
O1—C7—H19	113.5 (2)	C15—C16—H27	113.1 (3)
C8—C7—H19	106.94 (18)	N6—C16—H28	109.3 (3)
C9—C7—H19	105.68 (19)	C15—C16—H28	109.6 (3)
C7—C8—C10	120.30 (12)	H27—C16—H28	106.7 (2)
C7—C8—C11	120.84 (13)	O2—C17—H29	112.0 (2)
C10—C8—C11	118.84 (11)	O2—C17—H30	111.8 (3)
N5—C9—C7	112.7 (2)	H29—C17—H30	111.3 (3)
N5—C9—H20	107.4 (2)	O2—C17—H31	112.3 (3)
C7—C9—H20	109.9 (2)	H29—C17—H31	106.1 (3)
N5—C9—H21	109.7 (2)	H30—C17—H31	102.9 (2)
C7—C9—H21	110.8 (2)	O3—C18—H32	109.9 (3)
H20—C9—H21	106.0 (2)	O3—C18—H33	107.7 (3)
O2—C10—C8	115.41 (11)	H32—C18—H33	108.8 (3)
O2—C10—C13	124.17 (13)	O3—C18—H34	111.0 (3)
C8—C10—C13	120.42 (12)	H32—C18—H34	106.7 (2)
C8—C11—C12	120.22 (13)	H33—C18—H34	112.8 (3)
C8—C11—H22	117.19 (18)		

Symmetry codes: (i) 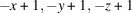; (ii) 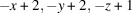; (iii) 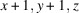; (iv) 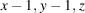.

**Table 2:** Hydrogen-bond geometry (Å, °)

*D*—H⋯*A*	*D*—H	H⋯*A*	*D*⋯*A*	*D*—H⋯*A*	Mulliken overlap	H-bond energy
N6—H37⋯O4	1.043	1.807	2.781	153.7	0.063	5.8
N6—H36⋯Cl38	1.054	2.070	3.094	163.2	0.088	
N6—H35⋯Cl38	1.047	2.181	3.159	154.6	0.077	
O1—H26⋯Cl38	0.993	2.159	3.146	172.1	0.064	35.6
N5—H23⋯Cl38	1.025	2.545	3.465	149.0	0.036	
C16—H28⋯Cl38	1.099	2.485	3.422	142.4	0.032	
C11—H22⋯O1	1.090	2.443	2.825	98.9	0.017	
C13—H24⋯O3	1.089	2.656	3.592	143.7	0.012	
C17—H31⋯O3	1.100	2.642	3.637	150.3	0.010	

**Table 3:** Experimental details

	midodrine
Crystal data	
Chemical formula	C_12_H_19_N_2_O_4_^+^·Cl^−^
*M*_r_	290.75
Crystal system, space group	Monoclinic, *P*2_1_/*c*
Temperature (K)	295
*a*, *b*, *c* (Å)	5.17893 (2), 8.25455 (3), 32.95227 (15)
β (°)	87.2465 (3)
*V* (Å^3^)	1407.08 (1)
*Z*	4
Radiation type	Synchrotron, λ = 0.46873 Å
μ (mm^−1^)	0.03
Specimen shape, size (mm)	Cylinder, 2.0 × 1.5

Data collection	
Diffractometer	11-BM, APS
Specimen mounting	Kapton capillary
Data collection mode	Transmission
Scan method	Step
2θ values (°)	2θ_min_ = 0.510, 2θ_max_ = 49.995, 2θ_step_ = 0.001

Refinement	
*R* factors and goodness of fit	*R*_p_ = 0.049, *R*_wp_ = 0.061, *R*_exp_ = 0.044, *R*(*F*^2^) = 0.03133, χ^2^ = 2.008
No. of parameters	96
No. of restraints	42
(Δ/σ)_max_	8.514

Computer programs: *GSAS-II* (Toby & Von Dreele, 2013 ▸).

## supplementary crystallographic information

### {[2-(2,5-Dimethoxyphenyl)-2-hydroxyethyl]carbamoyl}methanaminium chloride (midodrine) Crystal data

**Table:**

C_12_H_19_N_2_O_4_^+^·Cl^−^	*V* = 1407.08 (1) Å^3^
*M**_r_* = 290.75	*Z* = 4
Monoclinic, *P*2_1_/*c*	*D*_x_ = 1.373 Mg m^−^^3^
*a* = 5.17893 (2) Å	Synchrotron radiation, λ = 0.46873 Å
*b* = 8.25455 (3) Å	µ = 0.03 mm^−^^1^
*c* = 32.95227 (15) Å	*T* = 295 K
β = 87.2465 (3)°	cylinder, 2.0 × 1.5 mm

### {[2-(2,5-Dimethoxyphenyl)-2-hydroxyethyl]carbamoyl}methanaminium chloride (midodrine) Data collection

**Table:**

11-BM, APS diffractometer	Scan method: step
Specimen mounting: Kapton capillary	2θ_min_ = 0.510°, 2θ_max_ = 49.995°, 2θ_step_ = 0.001°
Data collection mode: transmission	

### {[2-(2,5-Dimethoxyphenyl)-2-hydroxyethyl]carbamoyl}methanaminium chloride (midodrine) Refinement

**Table:**

Least-squares matrix: full	96 parameters
*R*_p_ = 0.049	42 restraints
*R*_wp_ = 0.061	34 constraints
*R*_exp_ = 0.044	Weighting scheme based on measured s.u.'s
*R*(*F*^2^) = 0.03133	(Δ/σ)_max_ = 8.514
49486 data points	Background function: Background function: "chebyschev-1" function with 6 terms: 35.73(7), -4.92(12), -6.39(12), 3.98(12), -3.39(9), -0.15(7), Background peak parameters: pos, int, sig, gam: 1.580(20), 4.39(21)e3, 2.09(13)e3, 0.100, 5.557(6), 4.34(6)e3, 2.74(7)e3, 0.100,
Profile function: Finger-Cox-Jephcoat function parameters U, V, W, X, Y, SH/L: peak variance(Gauss) = Utan(Th)^2^+Vtan(Th)+W: peak HW(Lorentz) = X/cos(Th)+Ytan(Th); SH/L = S/L+H/L U, V, W in (centideg)^2^, X & Y in centideg 1.163, -0.126, 0.063, 0.000, 0.000, 0.002,	Preferred orientation correction: Simple spherical harmonic correction Order = 2 Coefficients: 0:0:C(2,-2) = -0.0581(18); 0:0:C(2,0) = 0.078(4); 0:0:C(2,2) = -0.0919(24)

### {[2-(2,5-Dimethoxyphenyl)-2-hydroxyethyl]carbamoyl}methanaminium chloride (midodrine) Fractional atomic coordinates and isotropic or equivalent isotropic displacement parameters (Å^2^)

**Table:**

	*x*	*y*	*z*	*U*_iso_*/*U*_eq_	
O1	1.0970 (4)	0.7465 (3)	0.57295 (6)	0.0323 (4)*	
O2	0.6632 (4)	0.9002 (2)	0.67443 (6)	0.0391 (5)*	
O3	1.3249 (4)	0.3806 (2)	0.68910 (6)	0.0391 (5)*	
O4	1.5002 (4)	1.0459 (3)	0.54902 (6)	0.0323 (4)*	
N5	1.0987 (4)	1.0938 (3)	0.57585 (7)	0.0323 (4)*	
N6	1.4661 (5)	1.2607 (3)	0.48827 (8)	0.0323 (4)*	
C7	0.9770 (5)	0.8305 (3)	0.60708 (6)	0.0323 (4)*	
C8	0.9847 (5)	0.7310 (3)	0.64575 (6)	0.0311 (5)*	
C9	1.1161 (5)	0.9912 (3)	0.61138 (8)	0.0323 (4)*	
C10	0.8228 (5)	0.7694 (3)	0.67930 (6)	0.0311 (5)*	
C11	1.1581 (5)	0.6018 (3)	0.64869 (7)	0.0311 (5)*	
C12	1.1601 (5)	0.5099 (3)	0.68409 (8)	0.0311 (5)*	
C13	0.8299 (6)	0.6787 (3)	0.71481 (7)	0.0311 (5)*	
C14	0.9978 (6)	0.5498 (3)	0.71712 (7)	0.0311 (5)*	
C15	1.2919 (5)	1.1156 (4)	0.54837 (8)	0.0323 (4)*	
C16	1.2304 (5)	1.2225 (4)	0.51286 (9)	0.0323 (4)*	
C17	0.4688 (6)	0.9328 (4)	0.70550 (9)	0.0391 (5)*	
C18	1.5119 (6)	0.3451 (4)	0.65719 (9)	0.0391 (5)*	
H19	0.76985	0.86202	0.60297	0.0388 (5)*	
H20	1.32549	0.96936	0.61586	0.0388 (5)*	
H21	1.04032	1.05789	0.63909	0.0388 (5)*	
H22	1.28492	0.57675	0.62242	0.0373 (6)*	
H23	0.93892	1.17104	0.57467	0.0388 (5)*	
H24	0.70477	0.71120	0.74223	0.0373 (6)*	
H25	1.00097	0.47574	0.74497	0.0373 (6)*	
H26	0.98954	0.64869	0.56984	0.0388 (5)*	
H27	1.09480	1.16753	0.49287	0.0388 (5)*	
H28	1.13894	1.33833	0.52442	0.0388 (5)*	
H29	0.35462	1.03463	0.69851	0.0469 (6)*	
H30	0.34623	0.82426	0.71243	0.0469 (6)*	
H31	0.55160	0.95960	0.73490	0.0469 (6)*	
H32	1.41042	0.31642	0.62801	0.0469 (6)*	
H33	1.61746	0.24085	0.66595	0.0469 (6)*	
H34	1.63568	0.44937	0.65061	0.0469 (6)*	
H35	1.60707	1.30873	0.50782	0.0388 (5)*	
H36	1.42630	1.35768	0.46795	0.0388 (5)*	
H37	1.53415	1.16293	0.47244	0.0388 (5)*	
Cl38	0.2652 (2)	0.55463 (11)	0.44075 (3)	0.0394	

### {[2-(2,5-Dimethoxyphenyl)-2-hydroxyethyl]carbamoyl}methanaminium chloride (midodrine) Atomic displacement parameters (Å^2^)

**Table:**

	*U*^11^	*U*^22^	*U*^33^	*U*^12^	*U*^13^	*U*^23^
Cl38	0.0457 (9)	0.0273 (9)	0.0446 (9)	0.0127 (16)	0.0036 (15)	−0.0002 (15)

### {[2-(2,5-Dimethoxyphenyl)-2-hydroxyethyl]carbamoyl}methanaminium chloride (midodrine) Geometric parameters (Å, º)

**Table:**

O1—C7	1.437 (2)	C15—O4	1.224 (2)
O1—H26	0.989 (2)	C15—N5	1.329 (2)
O2—C10	1.3741 (17)	C15—C16	1.5115 (19)
O2—C17	1.427 (3)	C16—N6	1.467 (3)
O3—C12	1.3814 (19)	C16—C15	1.5115 (19)
O3—C18	1.425 (3)	C16—H27	1.085 (3)
O4—C15	1.224 (2)	C16—H28	1.125 (3)
N5—C9	1.451 (2)	C17—O2	1.427 (3)
N5—C15	1.329 (2)	C17—H29	1.060 (3)
N5—H23	1.047 (2)	C17—H30	1.115 (3)
N6—C16	1.467 (3)	C17—H31	1.100 (3)
N6—H35	1.072 (3)	C18—O3	1.425 (3)
N6—H36	1.070 (2)	C18—H32	1.142 (3)
N6—H37	1.015 (3)	C18—H33	1.067 (3)
C7—O1	1.437 (2)	C18—H34	1.089 (3)
C7—C8	1.5181 (10)	H19—C7	1.118 (3)
C7—C9	1.519 (3)	H20—C9	1.116 (3)
C7—H19	1.118 (3)	H21—C9	1.121 (3)
C8—C7	1.5181 (10)	H22—C11	1.0811 (19)
C8—C10	1.3914 (18)	H23—N5	1.047 (2)
C8—C11	1.4010 (18)	H24—C13	1.119 (2)
C9—N5	1.451 (2)	H25—C14	1.1036 (19)
C9—C7	1.519 (3)	H26—O1	0.989 (2)
C9—H20	1.116 (3)	H26—Cl38^i^	2.1733 (10)
C9—H21	1.121 (3)	H27—C16	1.085 (3)
C10—O2	1.3741 (17)	H28—C16	1.125 (3)
C10—C8	1.3914 (18)	H29—C17	1.060 (3)
C10—C13	1.391 (2)	H30—C17	1.115 (3)
C11—C8	1.4010 (18)	H31—C17	1.100 (3)
C11—C12	1.3917 (18)	H32—C18	1.142 (3)
C11—H22	1.0811 (19)	H33—C18	1.067 (3)
C12—O3	1.3814 (19)	H34—C18	1.089 (3)
C12—C11	1.3917 (18)	H35—N6	1.072 (3)
C12—C14	1.383 (2)	H35—Cl38^ii^	2.1656 (10)
C13—C10	1.391 (2)	H36—N6	1.070 (2)
C13—C14	1.378 (2)	H36—Cl38^iii^	2.0529 (10)
C13—H24	1.119 (2)	H37—N6	1.015 (3)
C14—C12	1.383 (2)	Cl38—H26^i^	2.1733 (10)
C14—C13	1.378 (2)	Cl38—H35^ii^	2.1656 (10)
C14—H25	1.1036 (19)	Cl38—H36^iv^	2.0529 (10)
			
C7—O1—H26	104.5 (2)	C12—C11—H22	122.58 (19)
C10—O2—C17	118.16 (15)	O3—C12—C11	123.4 (2)
C12—O3—C18	118.2 (2)	O3—C12—C14	116.5 (2)
C9—N5—C15	123.69 (18)	C11—C12—C14	120.08 (12)
C9—N5—H23	117.7 (2)	C10—C13—C14	120.27 (14)
C15—N5—H23	117.5 (2)	C10—C13—H24	120.83 (18)
C16—N6—H35	108.7 (2)	C14—C13—H24	118.9 (2)
C16—N6—H36	109.0 (2)	C12—C14—C13	120.11 (13)
H35—N6—H36	104.9 (2)	C12—C14—H25	119.3 (2)
C16—N6—H37	111.8 (3)	C13—C14—H25	120.6 (2)
H35—N6—H37	111.9 (2)	O4—C15—N5	124.03 (18)
H36—N6—H37	110.3 (2)	O4—C15—C16	120.44 (17)
O1—C7—C8	111.60 (16)	N5—C15—C16	115.28 (18)
O1—C7—C9	107.8 (2)	N6—C16—C15	110.65 (17)
C8—C7—C9	111.2 (2)	N6—C16—H27	107.5 (3)
O1—C7—H19	113.5 (2)	C15—C16—H27	113.1 (3)
C8—C7—H19	106.94 (18)	N6—C16—H28	109.3 (3)
C9—C7—H19	105.68 (19)	C15—C16—H28	109.6 (3)
C7—C8—C10	120.30 (12)	H27—C16—H28	106.7 (2)
C7—C8—C11	120.84 (13)	O2—C17—H29	112.0 (2)
C10—C8—C11	118.84 (11)	O2—C17—H30	111.8 (3)
N5—C9—C7	112.7 (2)	H29—C17—H30	111.3 (3)
N5—C9—H20	107.4 (2)	O2—C17—H31	112.3 (3)
C7—C9—H20	109.9 (2)	H29—C17—H31	106.1 (3)
N5—C9—H21	109.7 (2)	H30—C17—H31	102.9 (2)
C7—C9—H21	110.8 (2)	O3—C18—H32	109.9 (3)
H20—C9—H21	106.0 (2)	O3—C18—H33	107.7 (3)
O2—C10—C8	115.41 (11)	H32—C18—H33	108.8 (3)
O2—C10—C13	124.17 (13)	O3—C18—H34	111.0 (3)
C8—C10—C13	120.42 (12)	H32—C18—H34	106.7 (2)
C8—C11—C12	120.22 (13)	H33—C18—H34	112.8 (3)
C8—C11—H22	117.19 (18)		

Symmetry codes: (i) −*x*+1, −*y*+1, −*z*+1; (ii) −*x*+2, −*y*+2, −*z*+1; (iii) *x*+1, *y*+1, *z*; (iv) *x*−1, *y*−1, *z*.

### (midodrine_midodrine_VASP) Crystal data

**Table:**

C_12_H_19_ClN_2_O_4_	*c* = 32.95150 Å
*M**_r_* = 290.75	β = 87.25°
Monoclinic, *P*2_1_/*c*	*V* = 1406.93 Å^3^
*a* = 5.17880 Å	*Z* = 4
*b* = 8.25410 Å	

### (midodrine_midodrine_VASP) Data collection

**Table:**

*h* = →	*l* = →
*k* = →	

### (midodrine_midodrine_VASP) Fractional atomic coordinates and isotropic or equivalent isotropic displacement parameters (Å^2^)

**Table:**

	*x*	*y*	*z*	*B*_iso_*/*B*_eq_	
O1	1.08609	0.75160	0.57288		
O2	0.66329	0.90588	0.67624		
O3	1.32010	0.37844	0.68884		
O4	1.49956	1.03863	0.54807		
N5	1.10117	1.10119	0.57712		
N6	1.46798	1.26472	0.48858		
C7	0.97486	0.83368	0.60772		
C8	0.98777	0.73521	0.64646		
C9	1.11992	0.99460	0.61196		
C10	0.82320	0.77413	0.68029		
C11	1.15908	0.60486	0.64894		
C12	1.16333	0.51097	0.68431		
C13	0.82942	0.68120	0.71575		
C14	0.99768	0.55011	0.71764		
C15	1.28930	1.11376	0.54791		
C16	1.23005	1.22586	0.51307		
C17	0.47051	0.93180	0.70822		
C18	1.50565	0.34561	0.65626		
H19	0.76985	0.86202	0.60297		
H20	1.32549	0.96936	0.61586		
H21	1.04032	1.05789	0.63909		
H22	1.28492	0.57675	0.62242		
H23	0.93892	1.17104	0.57467		
H24	0.70477	0.71120	0.74223		
H25	1.00097	0.47574	0.74497		
H26	0.98954	0.64869	0.56984		
H27	1.09480	1.16753	0.49287		
H28	1.13894	1.33833	0.52442		
H29	0.35462	1.03463	0.69851		
H30	0.34623	0.82426	0.71243		
H31	0.55766	0.96164	0.73709		
H32	1.41042	0.31642	0.62801		
H33	1.61746	0.24085	0.66595		
H34	1.63568	0.44937	0.65061		
H35	1.60707	1.30873	0.50782		
H36	1.42630	1.35768	0.46795		
H37	1.53415	1.16293	0.47244		
Cl38	1.26072	1.55497	0.44040		

### (midodrine_midodrine_VASP) Hydrogen-bond geometry (Å, º)

**Table:**

*D*—H···*A*	*D*—H	H···*A*	*D*···*A*	*D*—H···*A*
N6—H37···O4	1.043	1.807	2.781	153.7
N6—H36···Cl38	1.054	2.070	3.094	163.2
N6—H35···Cl38	1.047	2.181	3.159	154.6
O1—H26···Cl38	0.993	2.159	3.146	172.1
N5—H23···Cl38	1.025	2.545	3.465	149.0
C16—H28···Cl38	1.099	2.485	3.422	142.4
C11—H22···O1	1.090	2.443	2.825	98.9
C13—H24···O3	1.089	2.656	3.592	143.7
C17—H31···O3	1.100	2.642	3.637	150.3

## References

[bb1] Altomare, A., Cuocci, C., Giacovazzo, C., Moliterni, A., Rizzi, R., Corriero, N. & Falcicchio, A. (2013). *J. Appl. Cryst.* **46**, 1231–1235.

[bb2] Antao, S. M., Hassan, I., Wang, J., Lee, P. L. & Toby, B. H. (2008). *Can. Mineral.* **46**, 1501–1509.

[bb3] Bernstein, J., Davis, R. E., Shimoni, L. & Chang, N. L. (1995). *Angew. Chem. Int. Ed. Engl.* **34**, 1555–1573.

[bb4] Bravais, A. (1866). *Etudes Cristallographiques.* Paris: Gauthier Villars.

[bb5] Bruno, I. J., Cole, J. C., Kessler, M., Luo, J., Motherwell, W. D. S., Purkis, L. H., Smith, B. R., Taylor, R., Cooper, R. I., Harris, S. E. & Orpen, A. G. (2004). *J. Chem. Inf. Comput. Sci.* **44**, 2133–2144. 10.1021/ci049780b 15554684

[bb6] Crystal Impact. (2025). *DIAMOND.* Crystal Impact GbR, Bonn, Germany.

[bb7] Dassault Systèmes. (2025). *BIOVIA Materials Studio 2025*. BIOVIA, San Diego, CA, USA.

[bb8] David, W. I. F., Shankland, K., van de Streek, J., Pidcock, E., Motherwell, W. D. S. & Cole, J. C. (2006). *J. Appl. Cryst.* **39**, 910–915.

[bb9] Donnay, J. D. H. & Harker, D. (1937). *Am. Mineral.* **22**, 446–467.

[bb39] Dovesi, R., Erba, A., Orlando, R., Zicovich-Wilson, C. M., Civalleri, B., Maschio, L., Rérat, M., Casassa, S., Baima, J., Salustro, S. & Kirtman, B. (2018). *WIREs Comput. Mol. Sci.* **8**, e1360.

[bb10] Erba, A., Desmarais, J. K., Casassa, S., Civalleri, B., Donà, L., Bush, I. J., Searle, B., Maschio, L., Edith-Daga, L., Cossard, A., Ribaldone, C., Ascrizzi, E., Marana, N. L., Flament, J.-P. & Kirtman, B. (2023). *J. Chem. Theory Comput.* **19**, 6891–6932. 10.1021/acs.jctc.2c00958 PMC10601489 36502394

[bb11] Etter, M. C. (1990). *Acc. Chem. Res.* **23**, 120–126.

[bb38] Favre-Nicolin, V. & Černý, R. (2002). *J. Appl. Cryst.* **35**, 734–743.

[bb12] Friedel, G. (1907). *Bull. Soc. Française Minéral.* **30**, 326–455.

[bb13] Gatti, C., Saunders, V. R. & Roetti, C. (1994). *J. Chem. Phys.* **101**, 10686–10696.

[bb14] Groom, C. R., Bruno, I. J., Lightfoot, M. P. & Ward, S. C. (2016). *Acta Cryst.* B**72**, 171–179. 10.1107/S2052520616003954 PMC4822653 27048719

[bb15] Hirshfeld, F. L. (1977). *Theor. Chim. Acta* **44**, 129–138.

[bb16] Kabekkodu, S., Dosen, A. & Blanton, T. N. (2024). *Powder Diffr.* **39**, 47–59.

[bb17] Kaduk, J. A. (2002). *Acta Cryst.* B**58**, 370–379. 10.1107/s0108768102003476 12037358

[bb18] Kaduk, J. A., Crowder, C. E., Zhong, K., Fawcett, T. G. & Suchomel, M. R. (2014). *Powder Diffr.* **29**, 269–273.

[bb19] Kim, S., Chen, J., Cheng, T., Gindulyte, A., He, J., He, S., Li, Q., Shoemaker, B. A., Thiessen, P. A., Yu, B., Zaslavsky, L., Zhang, J. & Bolton, E. E. (2023). *Nucleic Acids Res.* **51**, D1373–D1380. 10.1093/nar/gkac956 PMC9825602 36305812

[bb20] Kresse, G. & Furthmüller, J. (1996). *Comput. Mater. Sci.* **6**, 15–50.

[bb21] Lee, P. L., Shu, D., Ramanathan, M., Preissner, C., Wang, J., Beno, M. A., Von Dreele, R. B., Ribaud, L., Kurtz, C., Antao, S. M., Jiao, X. & Toby, B. H. (2008). *J. Synchrotron Rad.* **15**, 427–432. 10.1107/S0909049508018438 18728312

[bb22] Macrae, C. F., Sovago, I., Cottrell, S. J., Galek, P. T. A., McCabe, P., Pidcock, E., Platings, M., Shields, G. P., Stevens, J. S., Towler, M. & Wood, P. A. (2020). *J. Appl. Cryst.* **53**, 226–235. 10.1107/S1600576719014092 PMC6998782 32047413

[bb23] Materials Design. (2024). *MedeA* Version 3.7.2. Materials Design Inc., San Diego, USA.

[bb24] MDI (2026). *JADE Pro*. Version 9.5. Materials Data, Livermore, USA.

[bb25] Motherwell, W. D. S., Shields, G. P. & Allen, F. H. (2000). *Acta Cryst.* B**56**, 857–871. 10.1107/S0108768100007230 11006562

[bb26] O’Boyle, N. M., Banck, M., James, C. A., Morley, C., Vandermeersch, T. & Hutchison, G. R. (2011). *J. Cheminform* **3**, 33. 10.1186/1758-2946-3-33 PMC3198950 21982300

[bb27] Peintinger, M. F., Oliveira, D. V. & Bredow, T. (2013). *J. Comput. Chem.* **34**, 451–459. 10.1002/jcc.23153 23115105

[bb28] Silk Scientific. (2013). *UN-SCAN-IT*. Version 7.0. Silk Scientific Corporation, Orem, USA.

[bb29] Singh, K. K., Desai, S. J. V. T. P. & Rupapara, M. L. (2022). United States Patent Application US 2022/0144754 A1.

[bb30] Spackman, P. R., Turner, M. J., McKinnon, J. J., Wolff, S. K., Grimwood, D. J., Jayatilaka, D. & Spackman, M. A. (2021). *J. Appl. Cryst.* **54**, 1006–1011. 10.1107/S1600576721002910 PMC8202033 34188619

[bb31] Stephens, P. W. (1999). *J. Appl. Cryst.* **32**, 281–289.

[bb34] Streek, J. van de & Neumann, M. A. (2014). *Acta Cryst.* B**70**, 1020–1032. 10.1107/S2052520614022902 PMC4468513 25449625

[bb32] Sykes, R. A., McCabe, P., Allen, F. H., Battle, G. M., Bruno, I. J. & Wood, P. A. (2011). *J. Appl. Cryst.* **44**, 882–886. 10.1107/S0021889811014622 PMC3246811 22477784

[bb33] Toby, B. H. & Von Dreele, R. B. (2013). *J. Appl. Cryst.* **46**, 544–549.

[bb35] Wang, J., Toby, B. H., Lee, P. L., Ribaud, L., Antao, S. M., Kurtz, C., Ramanathan, M., Von Dreele, R. B. & Beno, M. A. (2008). *Rev. Sci. Instrum.* **79**, 085105. 10.1063/1.2969260 19044378

[bb36] Wavefunction (2025). *Spartan ’24.* Version 1.3.1. Wavefunction Inc., Irvine, USA.

[bb37] Wheatley, A. M. & Kaduk, J. A. (2019). *Powder Diffr.* **34**, 35–43.

